# Synaptic E-I Balance Underlies Efficient Neural Coding

**DOI:** 10.3389/fnins.2018.00046

**Published:** 2018-02-02

**Authors:** Shanglin Zhou, Yuguo Yu

**Affiliations:** State Key Laboratory of Medical Neurobiology, School of Life Science and the Collaborative Innovation Center for Brain Science, Institutes of Brain Science, Center for Computational Systems Biology, Fudan University, Shanghai, China

**Keywords:** stimulus representation, information propagation, excitatory-inhibitory balance, sparse coding, energy efficiency

## Abstract

Both theoretical and experimental evidence indicate that synaptic excitation and inhibition in the cerebral cortex are well-balanced during the resting state and sensory processing. Here, we briefly summarize the evidence for how neural circuits are adjusted to achieve this balance. Then, we discuss how such excitatory and inhibitory balance shapes stimulus representation and information propagation, two basic functions of neural coding. We also point out the benefit of adopting such a balance during neural coding. We conclude that excitatory and inhibitory balance may be a fundamental mechanism underlying efficient coding.

## Introduction

Neural information coding is one of the central topics in neuroscience. The brain utilizes some features of action potential sequences (spike trains) to encode sensory and cognitive information. The algorithm operating within those features is called neural code. Half a century ago, Perkel and Bullock noted that a potential neural code must serve at least four functions: stimulus representation, interpretation, transformation, and transmission (Perkel and Bullock, 1968). In this review, we will mainly focus on the representation and transmission parts. For the other aspects of neural coding, please refer to other books (Rieke et al., 1997; Quiroga and Panzeri, 2013). Stimulus representation indicates that the neural activity should be altered by the stimulus properties needed to be coded, and therefore, the neural code can represent this stimulus (Perkel and Bullock, 1968; Kumar et al., 2010). Due to its basic role, neural representation has been extensively studied using experimental and theoretical approaches. Barlow in 1961 proposed a theoretical framework which hypothesized that the action potentials in the sensory neurons formed a neural code for efficiently representing sensory information. By efficient Barlow meant that the code minimized the number of neurons and spikes needed to represent an input signal. This is the origin of sparse coding or efficient neural coding (Barlow, 1961). Barlow's model treats the sensory pathway as a communication channel where neuronal spiking is an efficient code for representing sensory signals. The spiking code aims to maximize available channel capacity by minimizing the redundancy between representational units (Simoncelli and Olshausen, 2001). In addition, one of the major components of a typical neural code is reliable information transmission. The brain is highly modular, and a successful neural code should be able to be transmitted (propagated) from one module to another with high fidelity (Perkel and Bullock, 1968; Kumar et al., 2010). The transmission property of neural coding has also drawn significant attention recently (Diesmann et al., 1999; Kistler and Gerstner, 2002; van Rossum et al., 2002; Litvak et al., 2003; Vogels and Abbott, 2005; Kumar et al., 2008, 2010). The balance of excitatory and inhibitory synaptic membrane currents (E/I balance) received by a neuron underlying its spontaneous firing and/or responding to sensory inputs has been widely observed (van Vreeswijk and Sompolinsky, 1996; Brunel, 2000; Shu et al., 2003; Wehr and Zador, 2003; Zhang et al., 2003; Froemke et al., 2007; Murphy and Miller, 2009). Here, E/I balance generally refers to excitatory-inhibitory balance in terms of either overall global balance or temporal balance on a fine time scale. Global E/I balance refers to the bulk measurement of relative contributions of excitatory and inhibitory synaptic currents received by a specific neuron. It is called global E/I balance if across a range of spatio-temporal conditions of interest, the ratio between the synaptic excitation and inhibition is kept approximately constant (on a slow time scale). In some situations, even the measurement of firing rates of excitatory and inhibitory neurons or excitatory and inhibitory synaptic conductances received by a neuron can represent E/I balance for individual neurons within the cortical circuit (Shu et al., 2003; Southwell et al., 2010; Xue et al., 2014; Froemke, 2015). Temporal balance indicates that the relative magnitudes of excitatory and inhibitory synaptic currents are matched in a point-to-point manner on a fast time scale (Okun and Lampl, 2008; Froemke, 2015; Denève and Machens, 2016). Global E/I balance is often used to examine pathological or dysfunctional brain states, whereas temporal E/I balance can be used to examine the effect of synaptic correlation on spiking timing to sensory input and stimulus feature selectivity. Both global and temporal E/I balances enable cortical operation in a precise manner to represent sensory inputs. The disruption of the cortical E/I balance has been demonstrated to cause cognitive dysfunction, such as schizophrenia (Yizhar et al., 2011; Murray et al., 2014). Because the E/I balance may be the key structure underlying the neural code and cognition, multiple questions arise: (1) How is the E/I balance achieved? (2) Why does the neural system choose such a scenario to function? (3) How does the E/I balance evolve during neural plasticity and coding? Specifically, how does the E/I balance influence information representation and propagation across different areas?

Recently, more and more studies are conducted to answer these questions. Here, we briefly summarize the evidence for the existence of an E/I balance in the cortex and the mechanisms by which the E/I balance is achieved. We then review the experimental and computational development on the impact of the E/I balance on neural coding, especially the processes of stimulus representation and information propagation.

## E/I balance is ubiquitous in cortical circuits

Over the last decades, E/I balance has been found to exist in many situations including ongoing spontaneous activity, sensory-evoked activity, and storage of memories. Synaptic plasticity at both excitatory and inhibitory synapses is suggested to play a central role in balancing the excitatory and inhibitory inputs to a target cell during the training or learning process (Vogels et al., 2011; Yu et al., 2014). The level of the developed balance depends on the time scale of the correlation between the excitatory and inhibitory inputs to the cell, ranging from a global balance, either without a correlation or with a correlation at a slower time scale, to a fine-scale balance for strong correlations with a fast time scale.

Global balance is quantified by using global measures of excitatory and inhibitory synaptic currents, including measuring spontaneous or ongoing excitatory and inhibitory postsynaptic currents (mEPSC and mIPSC) and the field potential, which is considered a rough signature of the relative timing and magnitude of excitation and inhibition (Froemke, 2015). In fact, it is difficult to simultaneously measure excitatory and inhibitory current inputs on the same neuron. However, researchers can overcome this challenge by recording the excitatory and inhibitory currents separately from a target neuron by using the voltage-clamp recording technique. For voltage-clamp recordings, cells were progressively moved through a series of holding potentials, typically from −85 to +10 mV, in steps of 10 mV, and held at each potential for a few minutes to record several trials at each membrane potential. Briefly, average holding currents were constructed using at least 10 trials per holding potential, and used to construct current vs. voltage (*I*–*V*) plots. In cortical neurons, the reversal potential of EPSCs is around 0 mV, whereas for GABAA-receptor-mediated IPSCs, it is around −75 mV. The average synaptic currents reverse at around −35 mV (i.e., reverse potential E_synapse_) for cortical neurons. The electrode contained 2 M caesium acetate and 50 mM QX-314 to minimize the contribution of K and Na currents. The recorded synaptic current is defined as

(1) $$I_{syn}=g_{syn}(V-E_{syn})$$

where the total measured synaptic conductance *g*_*syn*_ is composed of the sum of the excitatory conductance (*g*_*e*_) and inhibitory conductance (*g*_*i*_) as follows:

(2) $$g_{syn}=g_{e}+g_{i}$$

In addition, the total synaptic current is the sum of the excitatory synaptic current *I*_*e*_ = *g*_*e*_(*V* − *E*_*e*_) and inhibitory synaptic current *I*_*i*_ = *g*_*i*_(*V* − *E*_*i*_),

(3) $$I_{syn}=g_{e}(V-E_{\text{e}})+g_{i}(V-E_{\text{i}})$$

where *E*_*e*_ = 0 mV is the reversal potential for excitatory synaptic current, and *E*_*i*_ = −75 mV is the reversal potential for inhibition for cortical neurons. To solve the above equations, we have

(4) $$g_{e}=\frac{g_{i}(E_{i}-E_{syn})}{E_{syn}-E_{e}},g_{i}=g_{syn}-g_{e}$$

**Figure 1:**
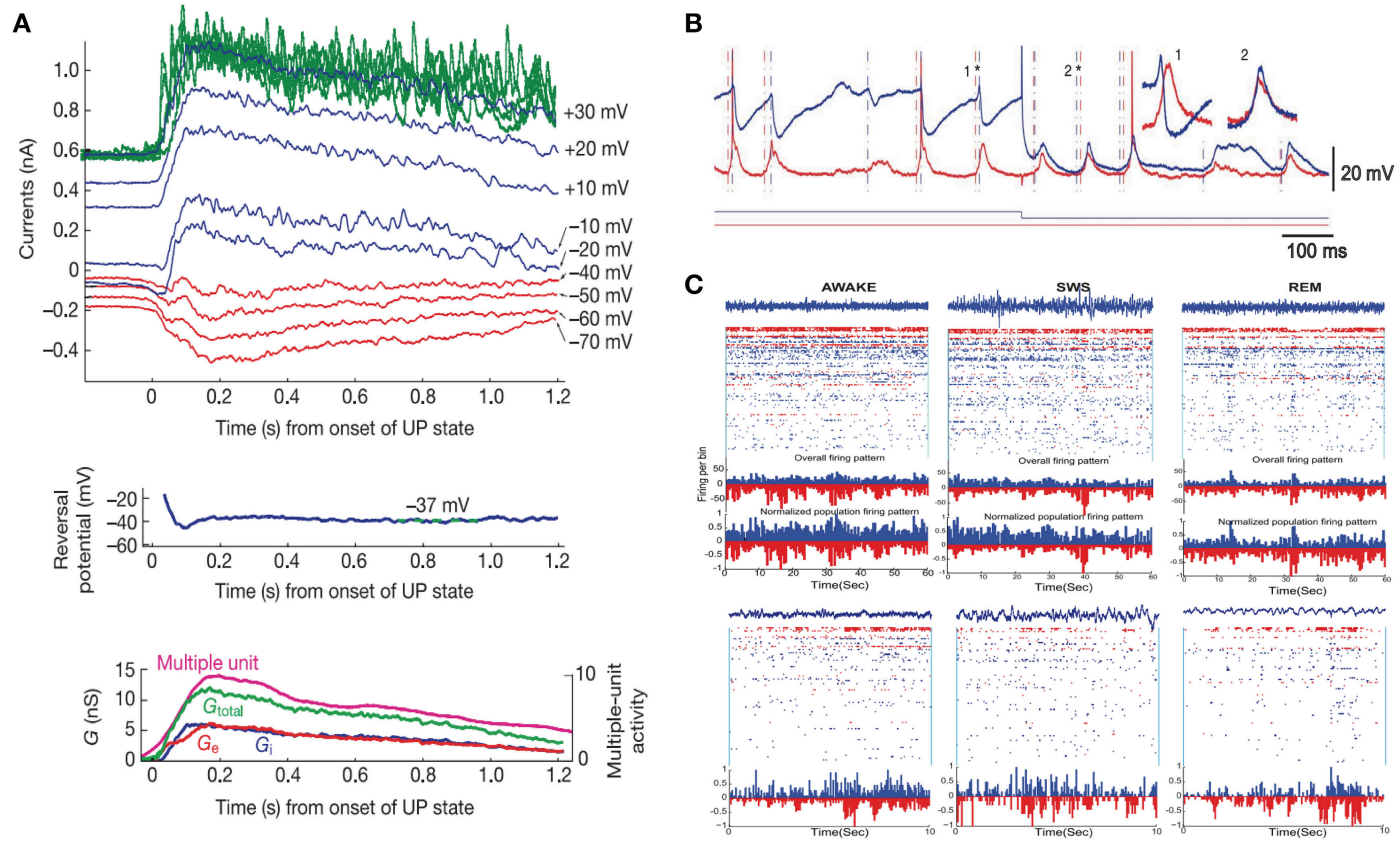
Experimental evidence of the E/I balance. **(A)** Average currents during the up state in recordings clamped at different membrane potentials from *in vitro* brain slices (top, red, and blule curves showing the average currents, the green curves showing the raw traces at +30 mV), the reversal potential of the average synaptic currents (middle), and additional conductances during the up state (bottom). Adapted from Shu et al. (2003). **(B)** Simultaneous *in vivo* recordings from two cortical cells. One cell (red) was continuously recorded in a hyperpolarized mode, and the other cell (blue) was switched between depolarized and hyperpolarized modes (current depicted below the traces). Dashed lines mark the onset of synaptic events. Insets show examples of two events (marked by asterisks). Adapted with permission from Okun and Lampl (2008). **(C)** Recordings in humans during awake (left), slow-wave sleep (SWS) (middle), and rapid-eye movement (REM) (right) states. Top row shows 60-s windows; bottom row shows a 10-s window of the same state. Putative inhibitory neurons (FS cells) are shown in red. Putative excitatory neurons (RS) are depicted in blue. At the top of each panel, a sample LFP trace (in blue) accompanies the spiking activity. Histograms show the overall activity of the RS (blue) and FS (red) cells. Adapted with permission from Dehghani et al. (2016).

This method helps to calculate the trial-by-trial average conductance of both currents (*g*_*e*_ and *g*_*i*_) (Shu et al., 2003; Haider et al., 2006; Monier et al., 2008). By using this method, Shu et al. found that the received synaptic conductance values of *g*_*e*_ and *g*_*i*_ were always balanced with a certain ratio during the up state generated by recurrent connection patterns in the *in vitro* brain slice (Figure 1A; Shu et al., 2003). Other experimental results also support the idea that the ratio of g_E_ and g_I_ of a given neuron remains constant across different conditions and in many systems (Wehr and Zador, 2003; Zhang et al., 2003; Haider et al., 2006; Xue et al., 2014). Additionally, many studies have demonstrated that the E/I balance still exists even when the system is driven by external inputs (Anderson et al., 2000; Martinez et al., 2002; Tan et al., 2004, 2011; Wilent and Contreras, 2005; Cardin et al., 2007; Wu et al., 2008; Tan and Wehr, 2009; Runyan et al., 2010; Liu et al., 2011). In fact, using *in vivo* whole-cell patch clamp, one can measure the excitatory or inhibitory conductance magnitude under different stimulus conditions. Instead of the classical tuning curve for firing rate vs. stimulus, one can plot the relation of conductance vs. stimulus (Anderson et al., 2000; Wehr and Zador, 2003; Zhang et al., 2003; Cardin et al., 2007; Runyan et al., 2010).

To understand the E/I balance on the fine time scale (the temporal balance), researchers have tried to simultaneously record the time series of both the excitatory and inhibitory currents and then obtain the correlation between them. Since adjacent neurons in the cortex generally receive strongly correlated synaptic inputs, researchers can record both excitatory and inhibitory currents separately and simultaneously, each in a single neuron in a pair of neighboring cells, and the correlation between the excitatory and inhibitory currents onto a single cell can be inferred from the correlation between the time series from the two cells (Okun and Lampl, 2008). Based on this method, researchers have found that the excitatory and inhibitory inputs from ongoing spontaneous activity or sensory-evoked activity are strongly correlated with one another, with inhibitory currents tracking excitatory currents closely with a few milliseconds of a delay (Figure 1B; Okun and Lampl, 2008). More evidence has also shown that a fine-scale E/I balance exists during oscillations in the gamma and beta frequencies (Atallah and Scanziani, 2009; Poo and Isaacson, 2009).

Recently, in an interesting study using *in vivo* recordings with dense multielectrodes in the neocortex of higher level mammals (including human and primate), Dehghani and colleagues found that excitatory and inhibitory ensembles are well-balanced and co-fluctuate instantaneously in all states of the wake-sleep cycle (wake, slow-wave sleep, and rapid-eye movement sleep) at different temporal scales (Figure 1C; Dehghani et al., 2016).

Beyond the temporal view of E/I balance, the spatial properties of E/I balance are also important in information processing. For instance, researchers found that local E/I imbalance coexisting with overall balance facilitates neural network creating novel features selectivity (Wu et al., 2006). However, we will mainly focus on temporal E/I balance in the following discussion.

## Mechanisms to achieve E/I balance

To achieve global E/I balance in a dynamic neural network, several theoretical studies have shown that a neural network needs to be equipped with the following properties: (1) neurons in the network should be connected sparsely (the number of connections per neuron should be much less than the total number of neurons in the network) and randomly; (2) the strength of the inhibitory connections should be high enough to balance network in which feedback excitation and inhibition could be canceled (van Vreeswijk and Sompolinsky, 1996, 1998; Brunel, 2000). Under such conditions, the average of the excitatory and inhibitory synaptic currents could be well-balanced, and the network dynamics could be stable. In such a balanced network, the membrane potentials and spike trains of the individual neurons may be highly uncorrelated (van Vreeswijk and Sompolinsky, 1996, 1998; Brunel, 2000). Beyond the two aspects mentioned above, synaptic plasticity may also play a vital role in the formation of the E/I balance (Froemke, 2015).

In contrast to requirements for global balance of a network, Renart et al. proposed a neural network with random and dense connections (with the number of connections per neuron comparable to the total number of neurons) to achieve a more fine-scale E/I balance (Renart et al., 2010) by tuning the synaptic conductances and connection structure. In such a network, the excitatory and inhibitory currents received by each neuron are strongly correlated on a fast time scale. If excitatory and inhibitory currents cancel each other, then the net input current will be highly random, resulting in highly variable neural responses. Boerlin et al. (2013) have demonstrated that both variable neural responses and balanced excitation/inhibition are necessary consequences of neural networks that represent information efficiently in their spike trains. However, the Boerlin model assumes instantaneous synapses (transmission without delays) and only achieves balance because of this assumption. Further work was allowed to relax this assumption and introduced realistic synapses with synaptic delays (Koren and Deneve, 2017). With realistic synapses, it is however required to fine-tune the parameters that weight the cost on spiking. Those parameters can be interpreted in biological terms as determining the excitability of the network.

Furthermore, a theoretical study proved that a network with synaptic plasticity of inhibitory synapses could evolve into a fine-scale E/I-balanced state with sparse connections (Vogels et al., 2011), and a later experimental study demonstrated the existence of this form of synaptic plasticity (D'amour and Froemke, 2015). Further information about inhibitory synaptic plasticity could be found in other good summaries (Kullmann et al., 2012; Sprekeler, 2017).

Beyond the theoretical work, experimental studies have provided some additional insights into the development of E/I balance (Dorrn et al., 2010; Sun et al., 2010; Tao et al., 2014; Froemke, 2015). For example, Liu et al. found that the ratio of the number of the excitatory and inhibitory synapses on the dendrites of cultured hippocampal neurons remained constant (Liu, 2004) along different developmental stages, which suggests that the E/I balance may be related to an anatomical basis. In addition, sensory experiences at different developmental stages may play important roles in shaping the final E/I balance level (Froemke, 2015).

## E/I balance and information representation

One of the fundamental functions of the neural systems is to represent the sensory information, a process termed neural coding, and make use of it for guiding action. Representing neural signals is the process of interpreting prominent features of external sensory inputs with individual or population neuronal activity. Experimental and theoretical studies have demonstrated that action potential generation is an energy-expensive process (Attwell and Laughlin, 2001; Alle et al., 2009; Yu et al., 2012). Therefore, efficient coding here can be either defined as maximal information coding with as few neurons and action potentials as possible (i.e., minimal cost) while not losing fidelity in the representation of certain stimulus features (equivalent to sparse coding as defined by Barlow, 1961) or minimized coding error during stimulus representation (Denève and Machens, 2016). Although there is no strict theoretical proof, the minimal coding error and information maximization or redundancy deduction are related in some aspects. Intuitively, coding error reduction means a decrease in noise information, which would increase the mutual information between the neural response and the input signal, thus increasing the information coding efficiency. Because E/I balance is ubiquitous in neural systems, there must be some strategic benefits of E/I balance for efficient representation.

Here, we summarize the evidence for this as follows.

### Irregular spike trains and global E/I balance

A typical well-known property of the firing pattern of an individual neuron recorded *in vivo* is its irregularity or stochasticity, which is similar to Poisson-like time sequences. Revealing how individual neurons establish such irregular firing patterns is important for understanding the network states with spontaneous firing and how such states could be used to represent stimulus inputs. In fact, it has been widely shown that irregular firing patterns could be achieved by a neuron with balanced excitatory and inhibitory synaptic inputs on multiple time scales (Shadlen and Newsome, 1994, 1998; van Vreeswijk and Sompolinsky, 1996; Amit and Brunel, 1997; Brunel, 2000). There is an intuitive explanation to why such a globally balanced network would lead to the irregular firing of a single neuron. Imagine that there is a neural network where each neuron is bombarded with noisy, Poisson-distributed synaptic inputs from both excitatory and inhibitory sources. When the excitatory input values exceed the inhibitory inputs, then the net mean positive input would depolarize the neuron to fire quasi-regularly. However, if the excitatory inputs and inhibitory inputs cancel each other out in a slow time scale without correlation on a fast time scale, then the membrane potential of each neuron would randomly cross the threshold dependent on the fast noise, resulting in a spiking pattern with a high level of irregularity (Denève and Machens, 2016).

Although such a network architecture would capture the irregularity of the neural firing pattern, the network behavior would become very sensitive to even a small perturbation due to its chaotic dynamics (Shadlen and Newsome, 1998; Brunel, 2000). This hypothesis suggests a low reliability of the neural network in response to sensory input, which makes such an E/I-balanced network represent stimulus features in a poor fidelity.

### Response sparseness and E/I balance

Action potential generation with actively pumping Na+ ions out of membrane for resting state recovery is considered to be energetically expensive (Siesjö, 1978), especially for cold-blooded animals (Yu et al., 2012). Lennie has estimated the cost of cortical computation processes in humans and suggests that only <1% of the total human brain can be substantially active concurrently (Lennie, 2003). However, this estimation is incorrect because his calculation is based on neuronal spiking costs in cold-blooded animals, but the spike process in warm-blooded animals is much more efficient in terms of energy cost (Alle et al., 2009; Howarth et al., 2012; Yu et al., 2012; Ju et al., 2016). Even so, the spiking process is still costly. Laughlin and Sejnowski suggested that to employ action potentials in neural coding in an energy-efficient manner, there may be evolutionary pressure to develop metabolically efficient neural codes (Laughlin, 2003). It was suggested that energy efficiency can be improved through the use of sparse coding (Field, 1994; Levy and Baxter, 1996).

**Figure 2:**
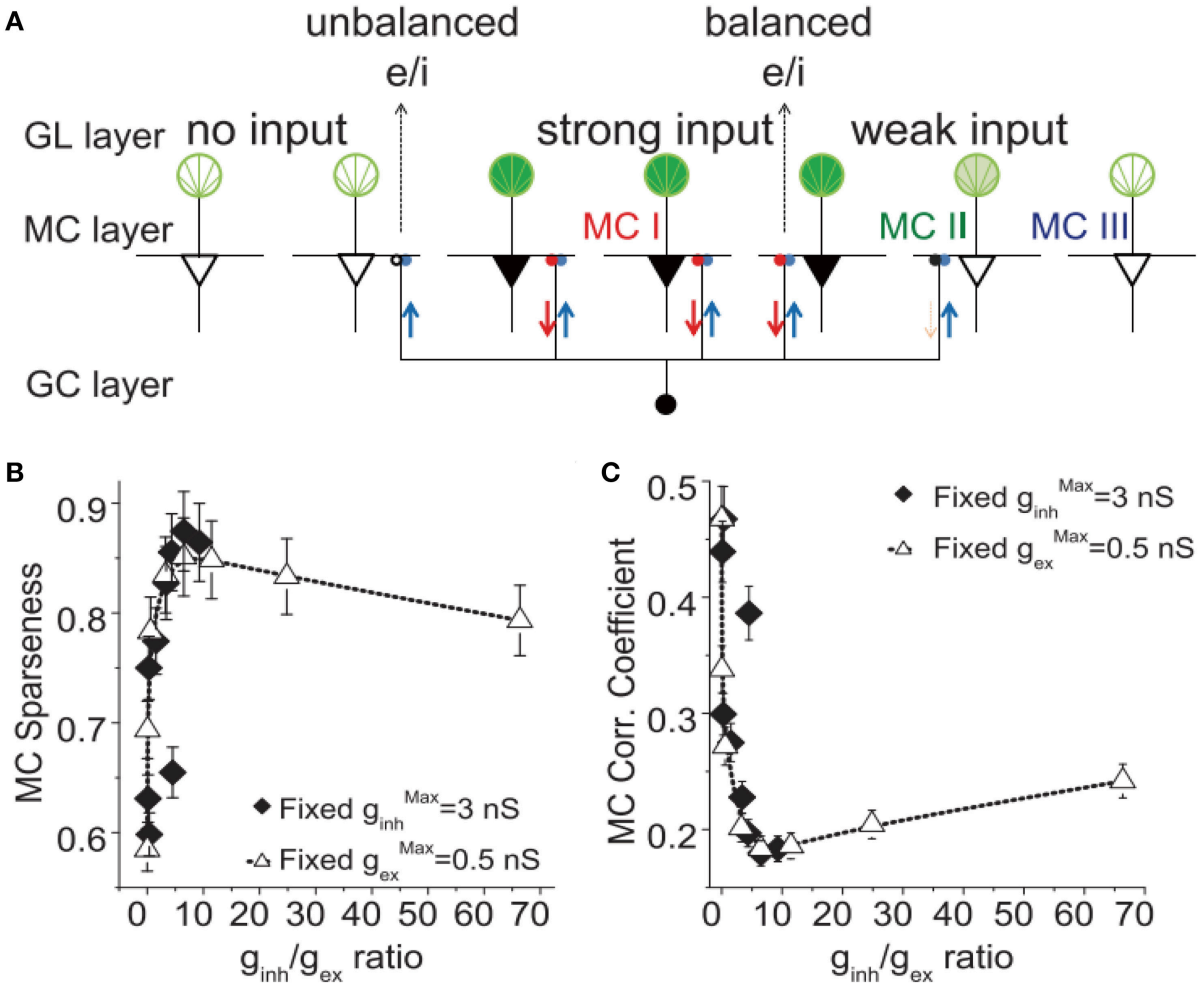
The correlation of firing sparseness and mitral cell spiking in a large-scale olfactory bulb model. **(A)** Schematic representation of balanced and unbalanced excitation and inhibition in the MC–GC circuit. Three activated middle MCs (solid black triangles) receive strong input from glomeruli (solid deep green color); through back-propagation of APs in their lateral dendrites, they distribute the excitation (red) through reciprocal synapses, activating lateral inhibition in the surrounding MCs through the reciprocal inhibitory synapses. This mode of excitation and inhibition is balanced, and these MCs are called MC type I. The activated GCs (small blue spheres) deliver lateral inhibition to other surrounding MCs with weak or no excitatory inputs, making their reciprocal synapses unbalanced. These MCs are called MC type II. MCs that do not receive lateral inhibition are MC type III. **(B)** The MC network sparseness level as a function of reciprocal inhibitory weight to excitation weight ratio g_inh_/g_ex_ for the cases of different ${\text{g}}_{\text{inh}}^{\text{Max}}$ with a fixed ${\text{g}}_{\text{ex}}^{\text{Max}}$ = 0.5 nS and different ${\text{g}}_{\text{ex}}^{\text{Max}}$ with a fixed ${\text{g}}_{\text{inh}}^{\text{Max}}$ = 0.3 nS. **(C)** Same in B but shows the mitral cell spiking correlation. Adapted from Yu et al. (2014).

Response sparseness is a phenomenon in which only a small fraction of cells in the network exhibit a transient response to an input signal (Vinje and Gallant, 2000). This implies a low-cost response to a stimulus, and such an energy-saving paradigm greatly extends the coding capacity of a large family of sensory inputs (Olshausen and Field, 1996; Dhawale et al., 2010; Wolfe et al., 2010; Koulakov and Rinberg, 2011). E/I balance has been found to be critical for generating response sparseness in several neural systems (King et al., 2013; Yu et al., 2013; Zhu and Rozell, 2015). Yu and colleagues implemented a large-scale olfactory bulb model with mitral and granule cells connected by dendro-dendritic synapses with regular LTP/LTD synaptic plasticity; they found that balanced excitation/inhibition in strongly activated mitral cells leads to a sparse representation of odorant inputs (Figure 2A; Yu et al., 2013, 2014). They further found that such a network with synaptic plasticity could always evolve into a sparsely oscillatory state to represent the input signal efficiently. During the evolving process, global synaptic excitation and inhibition gradually reach an optimal balance, with which the network produces firing patterns with the highest level of sparseness (Figure 2B; Yu et al., 2014). The optimal level of synaptic excitation and inhibition could produce the highest level of sparseness and decorrelation in the network response and reduce the energy cost (Nawroth et al., 2007). Yu and colleagues reported network simulation results indicating that higher sparseness is always associated with more decorrelated responses (Yu et al., 2014) and continuously learning new experience could further improve a network's sparse responses (Zhou et al., 2016). The decorrelated responses suggest a reduction in the redundancy of the coding scheme, thus resulting in efficient coding in an energy-saving manner. Therefore, in some previous publications, network response sparseness is also referred to as sparse coding. Such a coding scheme is similar to efficient coding in some aspects, but efficient coding is not necessarily accompanied by neural response sparseness.

Interestingly, the formation of response sparseness in such an olfactory bulb network does not depend on a specific type of synaptic plasticity, meaning either Hebbian or non-hebbian rules can both develop the network dynamics into sparseness during the training process (Migliore et al., 2010; Yu et al., 2013, 2014). In a recent work by Vogels et al. (2011), the sparsely connected network endowed with plasticity of inhibitory synapses could evolve to a sparse response to natural stimuli (Vogels et al., 2011). In addition, this type of network can accommodate synaptic memories with activity similar to the background activity; and same activity can be reactivated by external stimuli (Vogels et al., 2011).

Note that the above approaches assumed sparse network connection, i.e., neurons receive few connections K compared to the size of the network N, so that K < < N. Such a sparse connectivity usually leads to uncorrelated excitation and inhibition, resulting in random fluctuations as input to neurons within network. Indeed, achieving efficient representation of input signal does not require network to be sparsely connected. Recent theoretical works investigated network with dense connections. In such a scenario, excitation and inhibition received in a neuron are strongly correlated while neuronal output spike trains are highly uncorrelated. Such a balanced network could represent information with minimal coding error in their spikes (Boerlin et al., 2013). To ensure the network performance with optimal E/I balance, two general mechanisms are required: (1) synaptic plasticity is a necessary condition to adjust the tight balance of synaptic excitation and inhibition, which can efficiently mediate the delicate response tuning necessary to selectively filter intense sensory input, condensing it to the network-sparse responses (Finelli et al., 2008; Boerlin et al., 2013; Froemke, 2015), and (2) neurons fire a spike only if it improves the representation of dynamical variables. When a network satisfies these conditions, it evolves naturally to achieve the objective representation of a time-varying input with a minimum number of spikes with maximal efficiency (Boerlin et al., 2013; Denève and Machens, 2016). In the following work, they further revealed that the maximally efficient network is right at the transition from synchronous to asynchronous network states (Koren and Deneve, 2017). Moreover, there is a tight relationship between coding efficiency and energy efficiency; it was observed that there is a “sweet spot” at which the maximal coding efficiency coincides with a rather low number of spikes (Boerlin et al., 2013; Koren and Deneve, 2017). Beyond using dense connections to implement an E/I-balanced network, their work also proposes the possibility that irregular firing could be used to efficiently represent stimulus in a population code strategy (Boerlin et al., 2013; Koren and Deneve, 2017). Deneve and Machens proposed a spike code strategy at the population level to employ irregular spikings in efficient coding of input signals (Denève and Machens, 2016). In their theory, a non-zero input stimulus causes membrane depolarization (so-called decoding error) of individual neurons first in the population code. As soon as one of the neurons hits the spike thresholds and fires, the estimated decoding errors decrease through fast recurrent inhibition driven by the spiking neuron. This results in an efficient spike code at the population level through tight E/I balance. Indeed, the large-scale mitral-granule olfactory bulb model simulated by Yu and colleagues has implemented such a coding process for efficient representation of input odors (Yu et al., 2014).

Using single-compartment computational models with stochastic voltage-gated ion channels, Sengupta et al. (2013) calculated information content under either E/I-balanced or unbalanced conditions. They found that balanced synaptic currents evoke fewer spikes per second than the unbalanced conditions but with more information content in a single spike (bits/spike) in the balanced conditions. The total informative rate is similar in the two conditions (Sengupta et al., 2013). These results strongly support the hypothesis that E/I balance can promote both coding efficiency and energy efficiency.

Indeed, maximizing the ratio of the coding capacity to energy cost has been suggested to be one of the key principles chosen by the nervous system to evolve under selective pressure, and the metabolic energy efficiency demands of the nervous system could be sufficiently large to influence the design, function and evolution of the brain (Niven and Laughlin, 2008). A recent theoretical work revealed a general rule for population coding in which the neuronal number should be sufficiently large to ensure reliable information transmission that is robust to the noisy environment but small enough to minimize energy cost (Yu et al., 2016). Experiments in cortex cultures, anesthetized rats, and awake monkeys, as well as computer models, have shown that balanced excitation/inhibition (E/I) could lead to a critical dynamic of avalanches in the cortical neural network (Shew et al., 2009; Poil et al., 2012; Yang et al., 2012). Networks with different E/I balance could results in distinct network activity dynamics. Networks with low E/I balance show wave-like propagation over short distances. Networks with intermediate E/I balance often display patterns that are able to span long distances in the network. Networks with high E/I balance have high network activity, but have little spatial coherence in their activity patterns. The critical-state dynamics (which is characterized by the inverse power-law distribution of neuronal avalanches) of avalanches and oscillations jointly emerge in a neuronal network model when excitation and inhibition is well-balanced. Neuronal avalanche is generally defined as an active activity or event emerging from a silence state and ending with a silence or inactive state (Beggs and Plenz, 2003). The number of metastable states (Haldeman and Beggs, 2005) and the dynamic range to the input stimuli (Shew et al., 2009), as well as the information capacity and transmission (Beggs, 2008; Shew et al., 2011) of the cortical neural networks, could be maximized at the critical point (Shew et al., 2009; Poil et al., 2012; Yang et al., 2012). Here, information capacity means the potential or limits on maximum information that can be transferred or coded by a neural parameter (e.g., firing rate or spike timing) of neural systems, which could help us to understand how sensory and other information is being processed in the brain (Barlow, 1961; Bialek, 1987; Rieke et al., 1997). Of particular interest are usually the optimal conditions under which the information between stimuli and responses is maximized (Barlow, 1961; Atick, 1992).

The developed E/I balance within brain circuits during rest, learning, and memory states may be beneficial for the brain to maintain an optimal state based on the theory of criticality. A large E/I ratio leads to a super-critical state whereby the neurons are highly activated and spikes among neurons are highly correlated. However, a small E/I ratio leads to a sub-critical state whereby the overall neural activity level drops and the spikes among neurons are random and not correlated (Yang et al., 2012). For information processing, highly correlated spikes reduce entropy in the former case, and in the latter case, the reduced correlation increases entropy, but this increase is counteracted by the concurrent drop in total information, resulting in maximal information transmission at a moderate E/I ratio (Shew et al., 2011). However, energy expenditure increases monotonically as the E/I ratio increases due to the increasing overall neural activity level. Therefore, a relatively large information transmission while relatively low energy cost, is expected to be maximized around an optimal E/I ratio (Poo and Isaacson, 2009; Yu et al., 2014; Denève and Machens, 2016).

### Decorrelation and E/I balance

The correlations among the spiking trains of all individual neurons in a network in response to sensory input can either help or harm the information transfer (Averbeck et al., 2006). More specifically, if positive signal correlations (i.e., neurons with similar selectivities of stimulus features) are linked to positive noise correlation, this would harm the information transfer. On the contrary, neurons have opposite stimulus selectivities, positive noise correlation help the information transfer. In many cases, correlations will not influence the information transfer (see for example, a theoretical study by Moreno-Bote et al., 2014). To overcome the spiking correlation problem induced by correlated presynaptic input, Renart et al. (2010) built a densely connected neural network with excitatory and inhibitory currents canceling each other on a fast time scale (fine-scale balance). By using such mechanism, they showed that, theoretically, a fine-scale balanced network could generate an asynchronous state of population activity with a low mean spiking correlation despite correlated inputs (Renart et al., 2010). In the same study described above, Yu and colleagues found the E/I balance-induced sparse representation of odorant inputs was accompanied by a decorrelated state of mitral cell firing patterns, and the maximal decorrelation value existed at the optimal level for synaptic excitation and inhibition for the sparseness (Figure 2C; Yu et al., 2014). In another interesting experimental work, researchers manipulated the excitation/inhibition ratio (E/I ratio) to obtain an optimal E/I ratio that maximized the information capacity by trading off between a lower correlation state (induced by low E/I ratio) and moderate activity (induced by a relatively high E/I ratio) (Shew et al., 2011).

## E/I balance and information propagation

Because the brain is highly modular, and spiking activity may carry a lot of neural information, it is important that the spiking activity can be transmitted from one module to another with high fidelity. Indeed, Perkel and Bullock (1968) noted that one of the major components of a typical neural code should be the inclusion of reliable information transmission or information propagation. The identification of the conditions under which spiking activity can propagate with high fidelity has attracted the attention of many theoretical researchers in the recent decade (Diesmann et al., 1999; Kistler and Gerstner, 2002; van Rossum et al., 2002; Litvak et al., 2003; Kumar et al., 2008, 2010). Researchers usually address the propagation topic using a model of a cascade of neural assemblies in which a single neuron can participate at multiple levels (termed a feedforward network). To construct a more biologically oriented neural network, theoretical works tend to embed a feedforward sub-network into a larger recurrent neural network. However, neurons in the feedforward sub-network receive stronger correlated excitation than the rest of the recurrent network. This may destabilize the activity of the recurrent network. To solve this defect, Aviel et al. added inhibitory neurons into the subset network to balance the extra excitation (Aviel et al., 2003).

**Figure 3:**
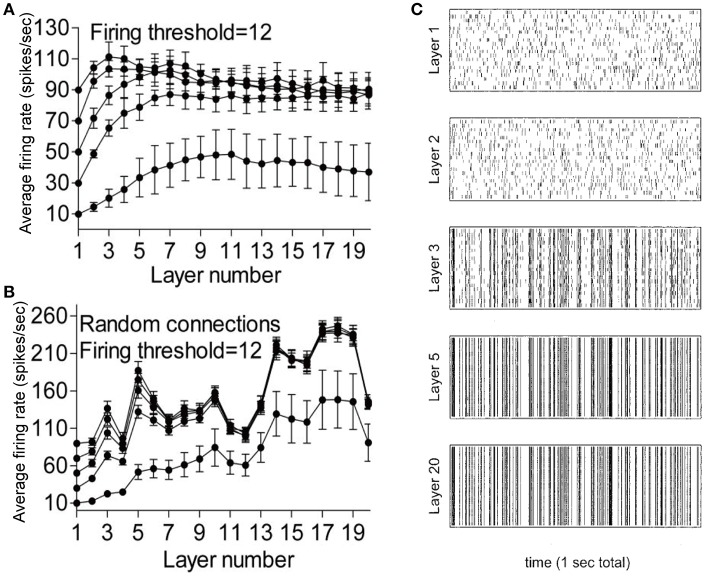
Propagation of firing rate in a multilayer feedforward network. **(A)** Average firing rate of different layers in the precisely balanced network. **(B)** Average firing rate of different layers in the feedforward network with small deviations from the precise balance. **(C)** Raster plot showing the firing pattern of excitatory neurons in different layers in a feedforward network with balanced firing rates between excitation and inhibition. Adapted with permission from Litvak et al. (2003).

Researchers have identified two modes of spiking activity propagation: the asynchronous mode (rate code, with information about the stimulus is carried and propagated in the firing rate of a neuron or the average of population activity; van Rossum et al., 2002; Litvak et al., 2003; Vogels and Abbott, 2009) and synchronous mode (temporal code, with information about the stimulus is carried and propagated by the precise timing of action potentials; Aertsen et al., 1996; Diesmann et al., 1999; Gewaltig et al., 2001; Litvak et al., 2003; Kumar et al., 2008). For a network with a feedforward configuration, the firing rates at each layer could be stabilized to a constant level after an initial increase (Figure 3A; Litvak et al., 2003). The network inhibition that precisely balanced with excitation plays a key role in modulating the mean firing rate level. Small deviations from the precise balance would result in a large fluctuation in the firing rate at each layer (Figure 3B; Litvak et al., 2003). Litvak et al. showed that the population synchrony could be formed after a few layers and then propagate stably through many layers in such a feedforward network with the excitation firing rate balanced with the inhibitory firing rate (Figure 3C; Litvak et al., 2003).

Beyond the fidelity of information propagation, the regulation of the spiking activity is also important for neural coding. A given module of the neural system has the potential to respond to several different signal pathways. To accomplish a single task, some mechanisms must exist to selectively block or boost some signal pathways. Recently, Vogels et al. showed that a detailed balance of excitation and inhibition in the target feedforward network group could be a potential gating mechanism; there information transmission can be gated “on” by adjusting the excitatory and inhibitory gains to upset this detailed balance (Vogels and Abbott, 2009).

## Conclusion

Stimulus representation and information propagation are two basic functions of neural coding. E/I balance, which is acknowledged as a fundamental paradigm for many brain functions, has been demonstrated to play a fundamental role in shaping the neural coding process. On one hand, the E/I balance can significantly increase the coding efficiency and energy efficiency to extend the coding capacity by promoting a sparse representation and signal decorrelation. Intuitively, an E/I ratio that is too high leads to excitatory dominance, resulting in high correlation (low level of coding efficiency) and activity (high level of energy consumed); however, an E/I ratio that is too low leads to suppressed activity with low information content. Therefore, the tradeoff between these two aspects requires the balance of the excitatory and inhibitory currents. On the other hand, based on recent theoretical studies, the E/I balance also plays a vital role in determining the fidelity of spiking activity propagation and gating of the multiple signal pathways. More experimental investigations are expected to test theoretical hypotheses and predictions in the near future. As discussed above, the implementations and functions of the two types of E/I balance, global balance and fine-scale balance, are different from one another. More studies are also needed to demonstrate the exact differences between the effects of these two types of balance on neural coding.

Here, we mainly discussed the roles of E/I balance in neural coding, while many additional studies have focused on the role of E/I balance in other brain functions, e.g., the formation of memories (Vogels et al., 2011; Lim and Goldman, 2013) and the information storage process. More studies are expected to clarify the effects of E/I balance in memory formation and examine how the information storage process benefits from the E/I balance.

## Author contributions

YY and SZ: designed research, wrote the paper, and performed research. All authors reviewed the manuscript.

### Conflict of interest statement

The authors declare that the research was conducted in the absence of any commercial or financial relationships that could be construed as a potential conflict of interest.
